# Rubinstein Taybi syndrome caused by *EP300* gene mutation: what we learned from two cases and literature review

**DOI:** 10.3389/fgene.2025.1588657

**Published:** 2025-07-02

**Authors:** Yefeng Wang, Xinghan Wu, Sha Zhao, Ningan Xu

**Affiliations:** ^1^ Department of Cardiology, The Affiliated Children’s Hospital of Xiangya School of Medicine, Central South University (Hunan Children’s Hospital), Changsha, Hunan, China; ^2^ Medical Genetics Department, The Affiliated Children’s Hospital of Xiangya School of Medicine, Central South University (Hunan Children’s Hospital), Changsha, Hunan, China; ^3^ Healthcare Center, The Affiliated Children’s Hospital of Xiangya School of Medicine, Central South University (Hunan Children’s Hospital), Changsha, Hunan, China

**Keywords:** Rubinstein-Taybi syndrome, EP300 gene, NSD1 gene, growth retardation, children

## Abstract

**Introduction:**

Rubinstein-Taybi syndrome is an extremely rare autosomal dominant genetic disease. The incidence of RSTS ranges from 1/100 000 to 125 000.

**Methods:**

We retrospectively reviewed the phenotype and genotype of two children who were diagnosed with RSTS in Hunan Province Children’s Hospital from January 2022 to December 2023. Clinical data of the children were collected. Whole-exome sequencing was performed on the children. The candidate variants were verified by Sanger sequencing in the pedigree, followed by pathogenicity analysis.

**Results:**

The main clinical presentations of the two cases were growth retardation, special facial features, and mild intellectual disability. Three mutations were detected by exome sequencing, all of which were sporadic mutations verified by Sanger sequencing. In case 1, pathological mutations were detected in *EP300* gene and *NSD1* gene. A heterozygous mutation c. 3934C>T (p. Arg1312Ter) was detected in exon 24 of EP300 gene. A heterozygous mutation c. 5843G>A (p. Arg1948 His) was detected in exon 18 of NSD1 gene. In case 2, a heterozygous mutation (c.2749C>T) (p. Gln917 *) was detected in exon 14 of *EP300* gene, which has not been reported in the literature so far. According to ACMG guidelines, this mutation was preliminarily determined to be pathogenic. Comparative analysis of phenotypic differences between the Chinese cohort and the Cohen JL and Fergelot P. cohorts revealed that arched eyebrows, downslanting palpebral fissures, and low-set ears were significantly more common in the Chinese population.

**Discussion:**

*EP300* gene c.2749C>T heterozygous mutation may be the genetic cause of Rubinstein Taybi syndrome. *EP300* gene combined with *NSD1* gene mutation may lead to atypical clinical presentations. These findings further enrich the variation spectrum of *EP300* gene.

## 1 Introduction

Rubinstein-Taybi syndrome (RSTS) is an extremely rare autosomal dominant genetic disease, first reported by Rubinstein and Taybi in 1963. The incidence of RSTS ranges from 1/100,000 to 125,000. It was reported mostly in Caucasians, and there was no difference between male and female populations. The main features of RSTS are growth retardation, microcephaly, facial deformities (e.g., lateral canthal slope, wide nasal bridge, and rostral nose), wide thumb and big toe, and mental and motor retardation (Milani et al., 2015). cAMP response element-binding protein (CREB) is encoded by chromosome 16p13.3 (OMIM #600140). *CREBBP* gene (Petrij et al., 1995)and chromosome 22q13.2 encodes E1A binding protein p300 (*EP300*) (OMIM #602700) *EP300* gene (Roelfsema et al., 2005). The variants of CREBBP gene account for 50%∼60%, which is called RSTS1 type; *EP300* gene mutations account for 8%∼10%, which is called RSTS type 2, but about 30% of patients have no pathogenic genes (Yang and Chen, 2023; Sun et al., 2021). This study reported the clinical phenotype and *EP300* gene variation of two children with RSTS, and explored the relationship between clinical phenotype and genotype of RSTS.

## 2 Study objects and methods

### 2.1 Research object

We retrospectively reviewed the phenotype and genotype of two children who were diagnosed with RSTS in Hunan Province Children’s Hospital from January 2022 to December 2023. The diagnosis of RSTS is established in a proband with characteristic clinical features (growth retardation, microcephaly, facial deformities wide thumb and big toe, and mental and motor retardation),with a heterozygous pathogenic variant (or likely pathogenic) or a chromosomal deletion involving one or more exons of the *EP300* gene. Children without genetic results and imaging data were not included in this study. This study was approved by the Ethics Committee of Hunan Province Children’s Hospital (HCHLL-2023-16), and the parents of the two children signed informed consent forms.

### 2.2 Research methods

#### 2.2.1 Clinical data collection

The basic information of the patients was collected through the electronic medical record system, including age at first diagnosis, clinical manifestations, growth and development history, maternal history and other general conditions, physical examination, laboratory examination results, gene sequencing results, treatment and prognosis, etc.

#### 2.2.2 Whole-exome sequencing (WES) and sanger validation

Whole-exome sequencing and Sanger sequencing validation were performed on the patients (conducted by Maijinuo Technology Co., Ltd.). Venous blood samples (2 mL each) were collected from the patients and their parents using EDTA anticoagulation. Genetic testing was carried out by Maijinuo Technology Co., Ltd. Exome capture was performed using the Agilent SureSelect method, followed by high-throughput sequencing on the Illumina platform. Sequencing data were analyzed using the GATK software, and variants were filtered using the TGex software. WES allowed the identification of putative causative mutations in *EP300* and *NSD1*, which were confirmed by Sanger sequencing. Primers were designed using the online primer design tool NCBI Primer-BLAST. PCR amplification products were purified with the Tiangen purification kit and sequenced on an ABI 3500DX Genetic Analyzer (Applied Biosystems, United States of America). Sequence alignment and analysis of the sequencing data were performed using SeqMan software.

The primer sequences of Sanger sequencing were as follows.

*EP300*- c.3934C>T-F1: GAT​TAG​CAT​GTT​CCC​TGC​ACT​C
*EP300*- c.3934C>T-R1: TTC​TGC​CAT​CTC​TCC​ACT​GTC
*EP300*- c.2749C>T-F1: GCT​CTT​CAT​CAG​AAT​TCA​CCC
*EP300*- c.2749C>T-R1: GGA​TTG​TGT​CCC​CTT​GTC​G
*NSD1*- c.5843G>A-F1: TGG​CAG​GGT​ACA​GAT​CTT​CA
*NSD1*- c.5843G>A-R1: ACC​TCC​TAC​ACA​GTG​ACC​ATG

#### 2.2.3 Bioinformatics analysis

The sequencing data were aligned to the human genome reference hg19 (GRCh37), and exome coverage and sequencing quality regions were evaluated. The raw Fastq data from whole-exome sequencing were analyzed using the ISoGenetic genetic variant interpretation system (integrating GATK, BWA mem, samtools, and Picard) and the CNVexon™ algorithm to identify copy number variations (CNVs), single nucleotide variants (SNVs), and small insertions/deletions (Indels). Detected genetic variants were annotated using databases including OMIM (http://www.omim.org/), ClinVar (https://www.ncbi.nlm.nih.gov/clinvar/), HGMD (http://www.hgmd.cf.ac.uk/ac/validate.php), gnomAD (http://gnomad.broadinstitute.org/about), and Ensembl (http://grch37.ensembl.org/index.html). Pathogenicity predictions for the variants were performed using SIFT and PROVEAN (http://provean.jcvi.org/index.php), PolyPhen-2 (http://genetics.bwh.harvard.edu/pph2/), MutationTaster (http://www.mutationtaster.org/), and CADD (https://cadd.gs.washington.edu/). Variants were classified into five categories based on the 2015 American College of Medical Genetics and Genomics (ACMG) guidelines and the Clinical Genome Resource (ClinGen) expert recommendations (Richards et al., 2015): ① pathogenic, ② likely pathogenic, ③ uncertain significance (VOUS), ④ likely benign, and ⑤ benign.

Protein sequences were retrieved from the UniProt database (https://www.uniprot.org), and wild-type and mutant protein structural models were generated using the SWISS-MODEL database (https://swissmodel.expasy.org/). Structural alterations in mutant proteins were further analyzed using PyMOL software.

#### 2.2.4 Literature search methods

A literature search was conducted using the keywords “Rubinstein-Taybi syndrome” and “*EP300* gene” in the CNKI, VIP, and Wanfang databases. Additionally, the keywords “Chinese Rubinstein-Taybi Syndrome” and “*EP300* gene” were used to search the PubMed database. The goal was to identify articles reporting the clinical characteristics of children with Rubinstein-Taybi syndrome (RSTS) caused by *EP300* gene mutations, with duplicate cases removed. Phenotypic differences between Chinese populations and cohorts of other ethnicities were compared. The search period spanned from the inception of each database to December 2024.

#### 2.2.5 Statistical methods

SPSS 20.0 software was used to analyze the data. Chi-square test or Fisher’s exact test was used to compare the clinical phenotypes. P < 0.05 was statistical difference.

## 3 Results

### 3.1 Clinical data

#### 3.1.1 Case 1

**FIGURE 1:**
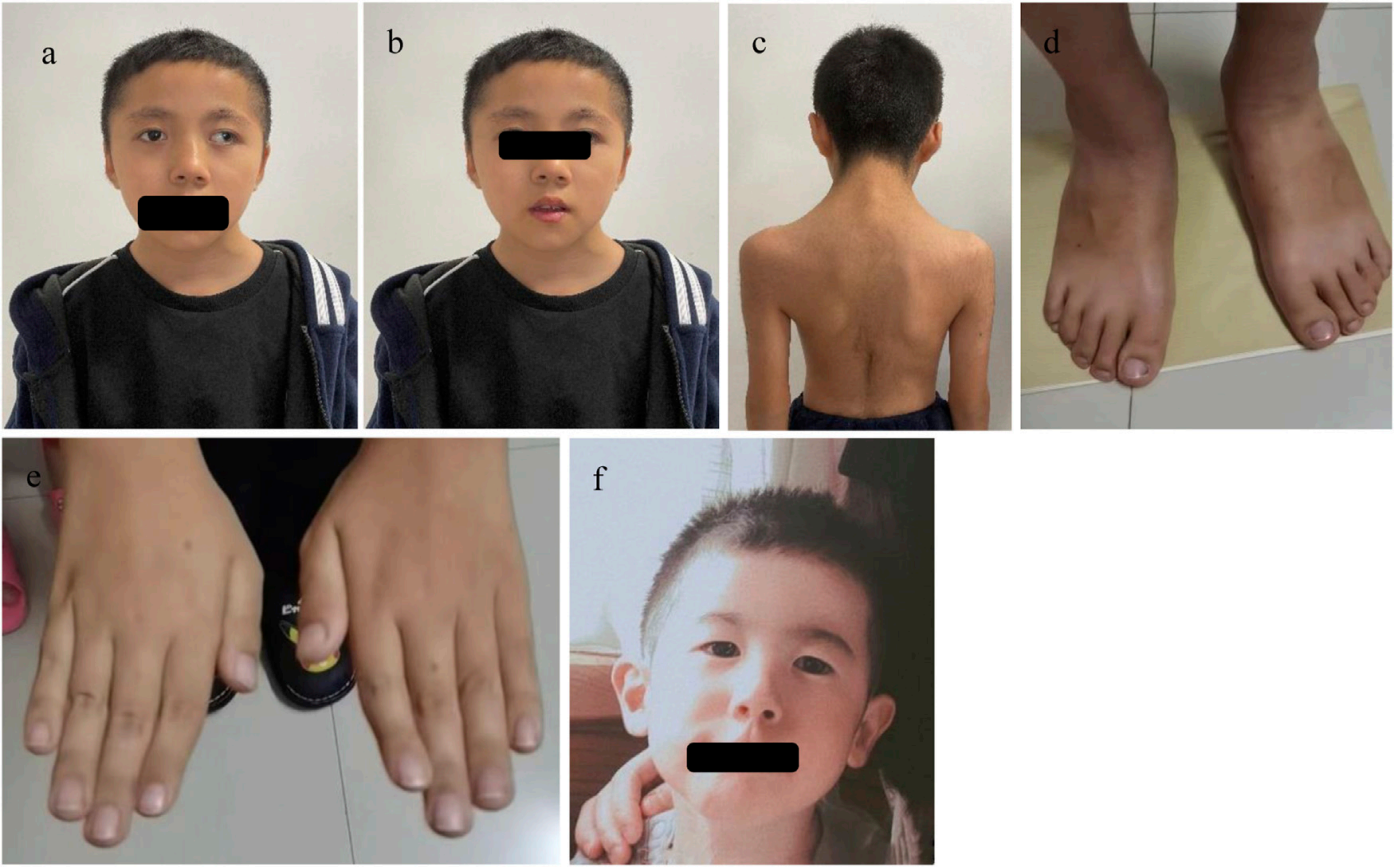
The facial features and other phenotypes of the cases: In Case 1, **(a)** shows microtia, broad nasal bridge, prominent nasal ridge, deviated nasal septum, down-slanting outer canthus, and exotropia; **(b)** shows thick eyebrows and micrognathia; **(c)** shows webbed neck, hypertrichosis, and low posterior hairline; **(d)** shows a slightly broad big toe; **(e)** shows no obvious broad thumb or fifth finger flexion deformity. In Case 2, **(f)** shows microcephaly, down-slanting outer canthus, and prominent nasal ridge.

**FIGURE 2:**
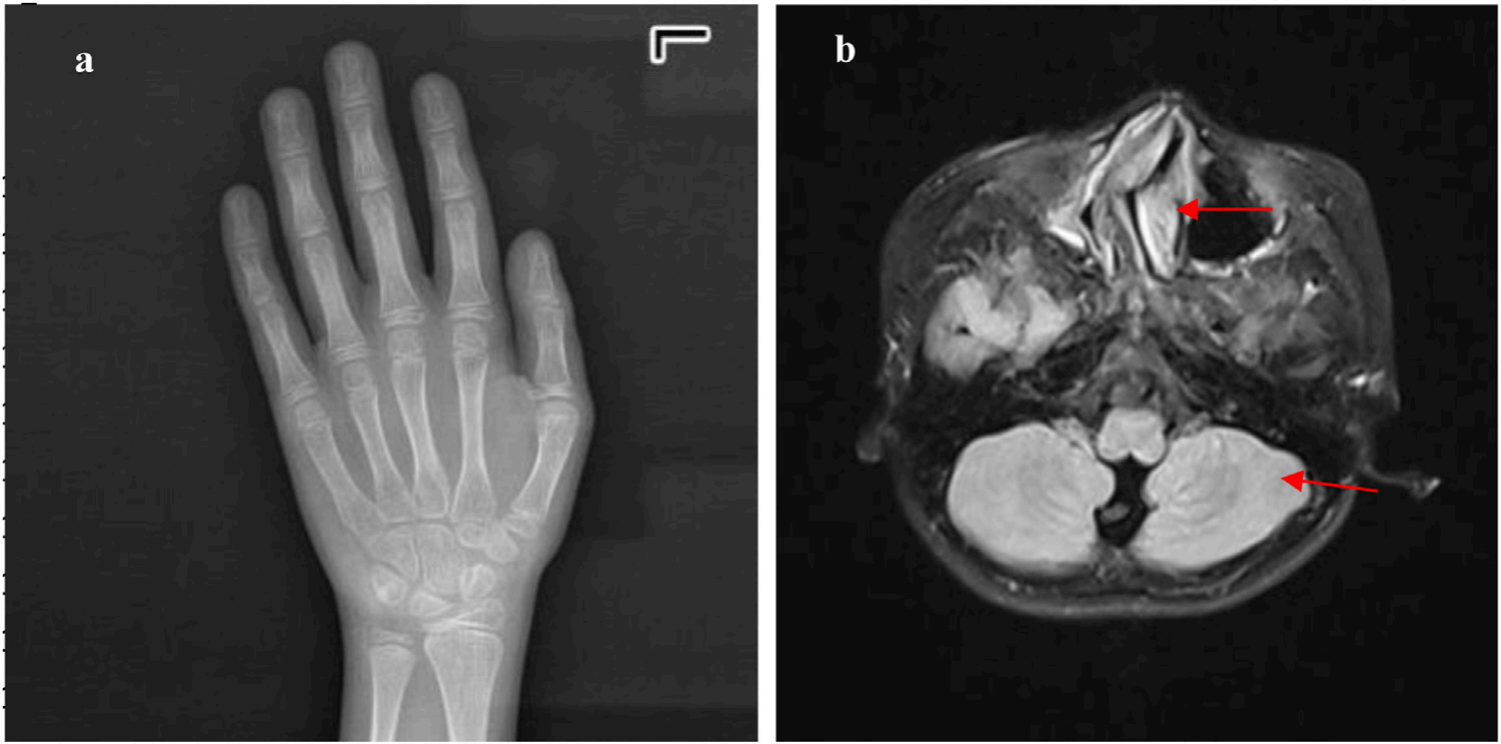
The metacarpal and phalangeal bones and cranial MRI of Case 1: **(a)** shows a bone age of 11 years, with an actual age of 10 years and 9 months, indicating the bone age is neither advanced nor delayed. **(b)** shows that the cranial MRI indicates slightly widened cerebellar sulci on the left side (red arrow), a markedly rightward deviation of the nasal septum in the middle (red arrow), and hypertrophy of the bilateral inferior nasal turbinates.

A 10-year and 7-month-old boy presented with growth retardation. He was the first child, born full-term with a birth weight of 3.0 kg (birth length unknown). He experienced hypoxia and asphyxia at birth. Developmental milestones: began conscious vocalizations (e.g., calling family members) at 1 year and 6 months, walked independently at 1 year and 8 months. Currently, his language expression lags behind peers, with poor motor coordination. He is in the third grade but performs poorly academically. Growth retardation in both height and weight was observed after birth, though the exact growth velocity remains unclear. No headaches, dizziness, visual field defects, polydipsia, or polyuria. VSD repair was performed at 1 year 3 months, followed by bilateral orchiopexy at age seven due to cryptorchidism. Family history: father’s height is 165 cm; mother’s height is 157 cm; mother has epilepsy managed with oxcarbazepine, with no seizures during pregnancy. Physical examination: weight is 24.2 kg (−1∼-2 SD), height is 128.6 cm (<-2 SD), head circumference is 48.5 cm (<-2 SD); proportionate build. Dysmorphic features: microcephaly, long eyelashes, downslanting palpebral fissures, small auricles, broad nasal bridge, prominent nasal ridge, micrognathia, thick eyebrows, exotropia (Figures 1a,b), webbed neck, hirsutism, low posterior hairline (Figure 1c). No broad thumbs (Figure 1e), slightly broadened great toes (Figure 1d). Tanner stage I genitalia. Investigations: blood/urine tests, biochemistry, and thyroid function are normal. Wechsler Intelligence Scale: IQ 65. Bone age (TW3 method): 11 years. Brain MRI: mildly widened left cerebellar sulci, significant right deviation of the nasal septum, and hypertrophic bilateral inferior turbinates (Figure 2).

#### 3.1.2 Case 2

A 2-year and 3-month-old boy presented with growth retardation. He was the first child, born preterm at 35 weeks via vaginal delivery with a birth weight of 2.13 kg (birth length unknown). Hospitalized in the neonatal unit due to preterm low birth weight; no intrauterine growth restriction noted prenatally. Family history: father’s height is 170 cm; mother’s height is 157 cm; unremarkable prenatal history. All developmental milestones are delayed compared to age-matched peers. Currently, the patient exhibits only minimal vocalizations, lacks meaningful words, and is unable to walk independently. Physical examination: height is 80 cm (<-2 SD), weight is 9.8 kg (<-2 SD), head circumference is 46.0 cm (<-2 SD); proportionate build. Dysmorphic features: microcephaly, downslanting palpebral fissures, micrognathia, broad nasal bridge (Figure 1f). No broad thumbs or great toes. Tanner stage I genitalia. Investigations: blood/urine tests, biochemistry, and thyroid function are normal. Gesell Developmental Schedules: Adaptive 62.7, Gross Motor 39.8, Fine Motor 54.2, Language 44.2, Personal-Social 41.8 (mild developmental delay). Brain MRI: No significant abnormalities.

### 3.2 Genetic test results

#### 3.2.1 Case 1

**FIGURE 3:**
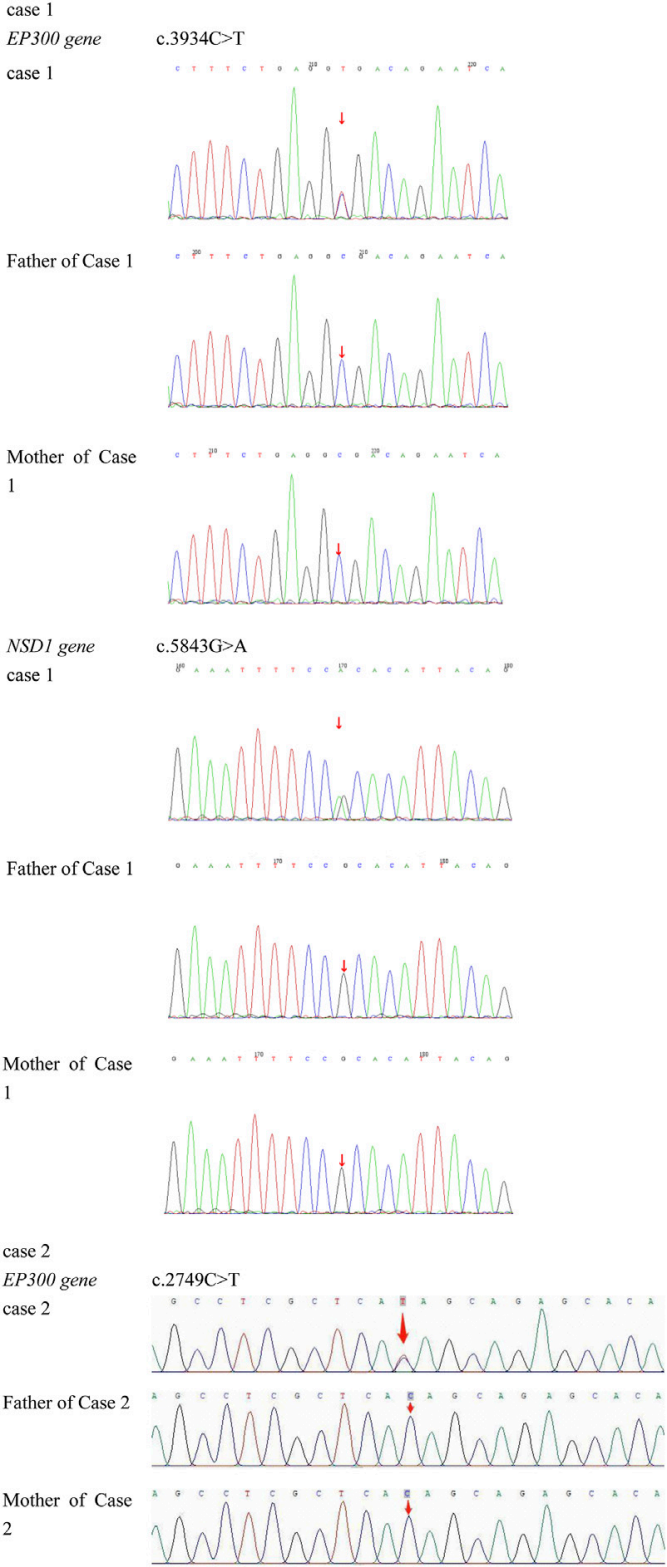
Case 1 carries a heterozygous mutation in exon 24 of the *EP300* gene: c.3934C>T (p.Arg1312Ter). Genetic testing of the parents showed no variation at this locus, confirming it as a *de novo* mutation. Additionally, Case 1 exhibits a heterozygous mutation in exon 18 of the *NSD1* gene: c.5843G>A (p.Arg1948His). Parental testing at this locus similarly revealed no variations, indicating another *de novo* mutation. Case 2 has a heterozygous mutation in exon 14 of the *EP300* gene: c.2749C>T (p.Gln917*). Testing of both parents demonstrated no genetic variation at this site, confirming it as a *de novo* mutation.

A heterozygous nonsense mutation in exon 24 of the *EP300* gene, c.3934C>T (p. Arg1312Ter), was identified. This mutation introduces a premature stop codon, resulting in a truncated protein. Both parents of the proband were tested and found to have no variation at this site, confirming it as a *de novo* mutation (Figure 3). This variant has been previously reported in the literature (Negri et al., 2015). According to the ACMG guidelines, this variant is preliminarily classified as pathogenic (PVS1 + PM2_Supporting + PS2 + PS4_Supporting).

#### 3.2.2 Case 2

A heterozygous mutation in exon 14 of the *EP300* gene, *c.2749C>T (p. Gln917) **, was identified. This mutation results in a premature stop codon at amino acid position 917, leading to a truncated protein and potential loss of function. This variant has not been reported in the literature or large-scale population frequency databases. Parental testing confirmed it as a *de novo* mutation (Figure 3). According to the ACMG guidelines, this variant is preliminarily classified as pathogenic (PVS1 + PM2_Supporting + PS2).

**FIGURE 4:**
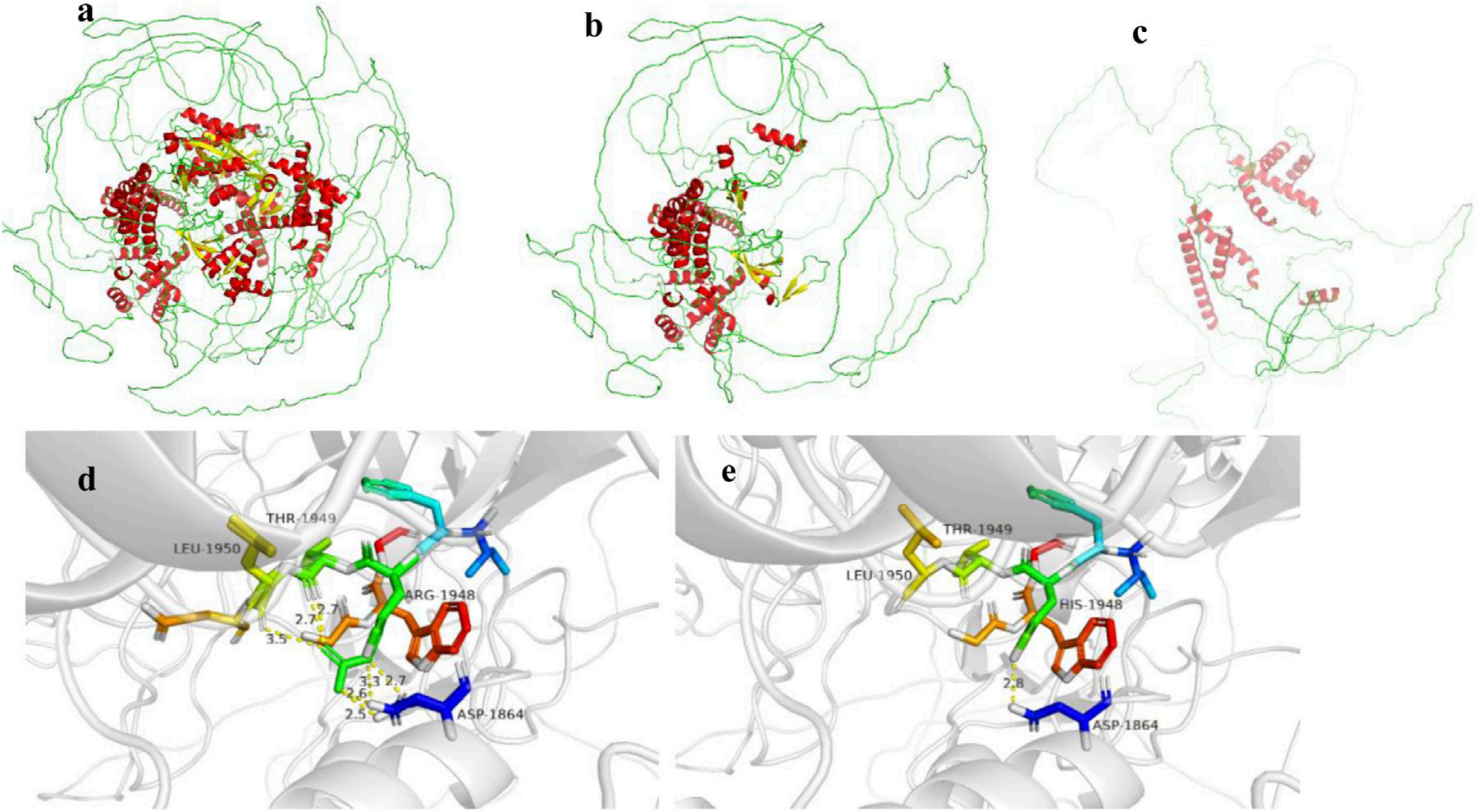
**(a–c)** show the *EP300* gene wild type, c.3934C>T (p.Arg1312Ter) mutation, and c.2749C>T (p.Gln917*) mutation, respectively. **(d,e)** show the *NSD1* gene wild type and c.5843G>A (p.Arg1948His) mutation, respectively.

Structural analysis using SWISS- MODEL (https://swissmodel.expasy.org/) online database and PyMOL software revealed that both mutations in the *EP300* gene result in truncated proteins, leading to loss of function (Figures 4b,c). Additionally, copy number variation (CNV) analysis was performed for Case 2, and no pathogenic CNVs were detected.

Case 1 also harbored a heterozygous mutation in exon 18 of the *NSD1* gene, c.5843G>A (p. Arg1948His), which is a missense mutation located in a mutation hotspot region (Figure 3). While mutations at the same position have been reported in the literature (Pohjola et al., 2012), the specific amino acid change (p. Arg1948His) has not been documented. Bioinformatics predictions using REVEL (score: 0.831), SIFT, PolyPhen-2, MutationTaster, and GERP + all indicated that this variant is deleterious. Parental testing confirmed it as a *de novo* mutation. This variant has not been reported in the literature, and its pathogenicity in the ClinVar database is classified as uncertain significance. Its frequency in the general population databases is 0.0001087. According to the ACMG guidelines, this variant is preliminarily classified as pathogenic (PM1 + PM5 + PP3_Moderate + PS2_P + PM2_P). Structural analysis using PyMOL software revealed that the 1948th amino acid of the *NSD1* protein is located in a β-sheet secondary structural domain. The substitution of arginine with histidine disrupts hydrogen bonds with aspartic acid at position 1864, threonine at position 1949, and leucine at position 1950, potentially leading to local structural instability and impaired protein function (Figure 4e).

### 3.3 Results of literature review

A total of 4 English articles and 4 Chinese articles meeting the criteria were identified. After removing duplicates and cases with incomplete information, six patients with clinical phenotypes and genotypes were collected. Including the two newly reported cases in this study, a total of 8 Chinese patients with Rubinstein-Taybi syndrome (RSTS) caused by *EP300* gene variants were included (Yang and Chen, 2023; Sun et al., 2021; Huang et al., 2023; Du et al., 2023; Bai et al., 2023; Yang et al., 2024) (details in Table 1). Among the eight patients, 1 case (12.5%) had a missense mutation, 3 cases (37.5%) had frameshift mutations, and 4 cases (50%) had nonsense mutations.

**TABLE 1:** Clinical phenotypes of Chinese patients with EP300 gene mutation RSTS.

Case	Gender	Age (yrs)	Chief complaint	Pregnancy abnormalities	Birth weight (g)	Mutation types	Exon	Mutated site	Amino acid	Short stature	Early feeding difficulties	Growth Retardation	Microcephalyi	Micrognathia	Thick eyebrows/arched eyebrows	Long eyelashes	High palatal arch	Grimace smile
1 Richards et al. (2015)	Male	11	Decreased vision	-	2,750	Frameshift mutation	21	c.3714_3715del	p.Leu1239Glyfs*3	-	-	Mild	+		+/+	-	NA	-
2 Negri et al. (2015)	Male	0.7	Pneumonia	-	2,250	Nonsense mutation	22	c.3750C>A	p.Cys1250*	-	-	Mild	+	NA	NA	NA	NA	NA
3 Negri et al. (2015)	Male	10	Fever	-	NA	Missense mutation	22	c.1889A>G	p.Tyr630Cys	-	+	Mild	NA	NA	NA	NA	NA	NA
4 Pohjola et al. (2012)	Male	9.3	Stunting	Preeclampsia	3,100	Nonsense mutation	20	c.3604G>T	p.E1202*	-	+	Moderate	+	+	+/+	+	+	+
5 Yang and Chen (2023)	Female	4	Recurrent respiratory tract infection	Preeclampsia	3,100	Frameshift mutation	14	c.2499dupG	p.Pro834Alafs*4	-	-	Mild	-	+	+/+	+	-	-
6 Sun et al. (2021)	Male	0.6	Stunting	-	2,350	Frameshift mutation	30	c.4853_4854del	p.Leu1618*	+	+	Mild	+	NA	−/+	-	NA	-
7	Male	10	Short stature	-	3,000	nonsense mutation	24	c.3934C>T	p.Arg1312Ter	+	+	Mild	+	+	+/+	+	+	-
8	Male	2 0.2	Stunting	Premature	2,130	Nonsense mutation	14	c.2749C>T	p.Gln917*	+	-	Mild	+	+	−/+	-	-	-

Case	Lateral canthus inferior oblique	Low ear position	Convex nasal ridge	Inferior nasal column	Wide/Angled Thumbs	Wide/Angled Big Toe	Syndactyly/polydactyly	Hirsutism	Central nervous system abnormalities	Ocular abnormalities	Cardiovascular abnormalities	Genitourinary abnormalities	Gastrointestinal abnormalities	Recurrent respiratory tract infection	Other
1	+	+	-	-	+/−	+/−	−/−	-	NA	Early onset glaucoma	-	-	-	+	obesity
2	+	NA	NA	NA	NA	NA	NA	NA	NA	NA	patent ductus arteriosus	-	NA	+	hypothyroidism Hearing loss, adrenal insufficiency, immune deficiency
3	NA	NA	NA	NA	+/−	+/−	−/−	NA	NA	NA	NA	hydrocele	NA	+	immunodeficiency
4	+	+	-	-	+/−	+/−	−/−	+	autistic behavior	trichiasis	-	concealed penis, hydrocele		-	Attention deficit and hyperactivity disorder
5	+	-	-	-	−/−	−/−	−/+	+	NA	-	-	-	-	+	immunodeficiency
6	+	+	+	-	+/−	+/−	+/−	-	Delayed myelination, bilateral lateral ventricle and third ventricle widening	-	-	-	-	-	
7	+	+	+	+	+/−	+/−	−/−	+	The left cerebellar sulcus is slightly wider, the middle part of nasal septum is obviously deviated to the right, and the inferior turbinate is thickened on both sides	Cataract, strabismus	ventricular septal defect	cryptorchidism	-	-	
8	+	-	-	-	−/−	−/−	−/−	-	-	-	-	-	-	-	

-: No phenotype; +: Phenotype present; NA: phenotype not described.

**FIGURE 5:**
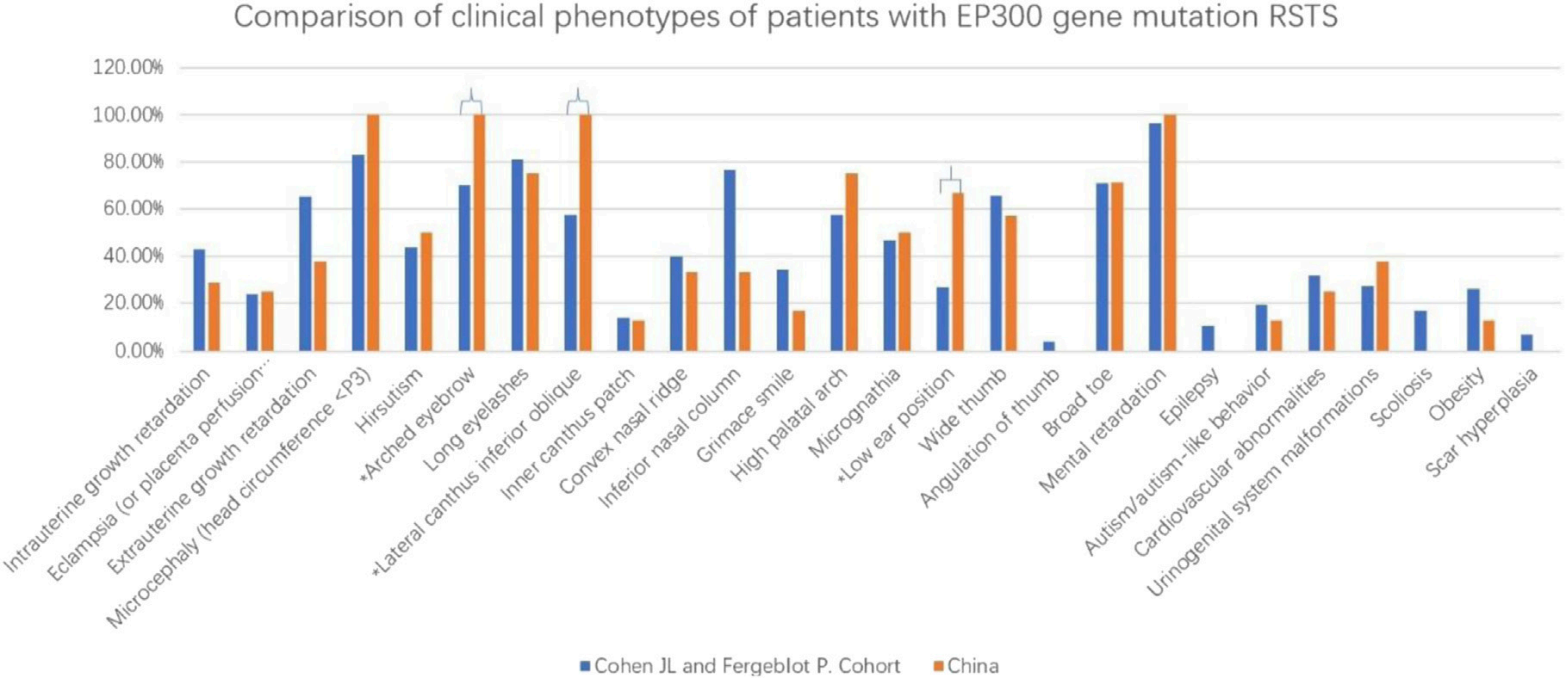
This figure revealed that arched eyebrows, downslanting palpebral fissures, and low-set ears were significantly more common in the Chinese population. *p < 0.05.

Comparative analysis of phenotypic differences between the Chinese cohort and the Cohen JL and Fergelot P. cohorts revealed that arched eyebrows, downslanting palpebral fissures, and low-set ears were significantly more common in the Chinese population, with statistical significance. Phenotypes such as angulated thumbs, severe intellectual disability, epilepsy, scoliosis, and keloid formation were not reported in the Chinese population (details in Figure 5; Table 2).

**TABLE 2:** Comparison of clinical phenotypes of patients with EP300 gene mutation RSTS.

Clinical Phenotypes	Cohen JL and Fergeblot P cohort			China		
	Positive	Total number	Proportion	Positive	Total number	Proportion
Intrauterine growth retardation	30	70	42.9%	2	7	28.6%
Eclampsia (or placenta perfusion disorder)	20	84	23.8%	2	8	25.0%
Extrauterine growth retardation	51	78	65.4%	3	8	37.5%
Microcephaly (head circumference < P3)	68	82	82.9%	7	7	100.0%
Hirsutism	31	71	43.7%	3	6	50.0%
Arched eyebrow	54	77	70.1%	6	6	100.0%^a^
Long eyelashes	56	69	81.2%	3	4	75.0%
Lateral canthus inferior oblique	43	75	57.3%	7	7	100.0%^a^
Inner canthus patch	11	81	13.6%	1	8	12.5%
Convex nasal ridge	29	73	39.7%	2	6	33.3%
Inferior nasal column	56	73	76.7%	2	6	33.3%
Grimace smile	23	67	34.3%	1	6	16.7%
High palatal arch	38	66	57.6%	3	4	75.0%
Micrognathia	34	73	46.6%	4	8	50.0%
Low ear position	20	75	26.7%	4	6	66.7%^a^
Wide thumb	52	79	65.8%	4	7	57.1%
Angulation of thumb	3	78	3.8%	0	7	0.0%
Broad toe	56	79	70.9%	5	7	71.4%
Intellectual disability	77	80	96.3%	8	8	100.0%
Severe	5	58	8.6%	0	8	0.0%
Moderate	12	58	20.7%	1	8	12.5%
Mild	41	58	70.7%	7	8	87.5%
Epilepsy	8	79	10.1%	0	8	0.0%
Autism/autism-like behavior	15	78	19.2%	1	8	12.5%
Cardiovascular abnormalities	24	76	31.6%	2	8	25.0%
Urinogenital system malformations	20	74	27.0%	3	8	37.5%
Scoliosis	13	78	16.7%	0	8	0.0%
Obesity	21	81	25.9%	1	8	12.5%
Scar hyperplasia	5	78	6.4%	0	8	0.0%

^a^ There was a statistical difference between the two groups p < 0.05.

## 4 Discussion

Rubinstein-Taybi Syndrome (RSTS) is a genetic disorder affecting multiple systems. Clinically, it is also referred to as “Broad Thumb-Hallux Syndrome”. Its main features include craniofacial abnormalities (e.g., microcephaly, thick hair, low hairline, arched/thick eyebrows, downslanting palpebral fissures, epicanthal folds, high-arched palate, micrognathia), skeletal abnormalities (e.g., broad thumbs/halluces, clinodactyly, scoliosis), short stature, and intellectual disability. Additionally, it may involve abnormalities in the respiratory, gastrointestinal, cardiovascular, nervous, and genitourinary systems (Van Gils et al., 2021).

In this study, both patients with *EP300* gene mutations exhibited short stature and dysmorphic features (e.g., microcephaly, downslanting palpebral fissures, micrognathia, broad nasal bridge), but no typical “grimacing smile” or broad/angulated thumbs. Their intellectual development was mildly delayed, consistent with previous reports. Case 1 had additional features such as webbed neck, hirsutism, and broad halluces, along with structural anomalies like cryptorchidism, ventricular septal defect, strabismus, and deviated nasal septum, while Case 2 lacked these phenotypes.

Pathogenic mutations in the *CREBBP* and *EP300* genes can cause RSTS. The encoded proteins, *EP300* and *CREBBP*, share high sequence similarity in the bromodomain, cysteine-histidine-rich region, and histone acetyltransferase (HAT) domain. Both play critical roles in embryonic development, growth control, and homeostasis by coupling chromatin remodeling with transcription factor recognition. Mutation types include intragenic deletions, whole-gene deletions, frameshift mutations, nonsense mutations, missense mutations, splice-site mutations, and duplications. Currently, most studies suggest no clear correlation between the severity or specificity of clinical phenotypes and the type of single-gene variant (Roelfsema and Peters, 2007; Kuai, 2023; Cohen et al., 2020).

*EP300* gene mutations account for 8%–11% of RSTS cases. The *EP300* protein, encoded by the *EP300* gene, functions as a histone acetyltransferase involved in chromatin remodeling and transcriptional regulation, which are essential for cell proliferation and differentiation. Additionally, *EP300* mediates cAMP gene regulation by specifically binding to phosphorylated CREB protein (Dyson and Wright, 2016). Previous reports indicate that patients with *EP300* variants often exhibit milder clinical manifestations compared to those with *CREBBP* variants or chromosome 16p13.3 abnormalities. The characteristic “grimacing smile” is less common in *EP300* variant patients, and features such as broad thumbs and angulated thumbs are rare. Intellectual impairment in *EP300* variant patients is typically milder, often presenting as mild to moderate intellectual disability or normal intelligence with learning difficulties (Fergelot et al., 2016).

Analysis of ClinVar database entries for pathogenic or likely pathogenic variants revealed that missense mutations in the *EP300* gene account for 18.8% (35/186), while loss-of-function variants (nonsense, frameshift, splice-site, and start codon loss) account for 76.3% (142/186). This distribution is similar to that observed in Chinese patients. The two newly reported *EP300* mutations in this study are truncating variants, which alter protein length, leading to loss of function, impaired transcription, and disrupted cell proliferation and differentiation, resulting in a range of clinical manifestations.

Case 1 also harbored a pathogenic variant in the *NSD1* gene, which is rare given that both variants were *de novo*. The parents were not of advanced age, and the mother took folic acid before and during pregnancy. The mutations may be associated with the mother’s long-term use of antiepileptic drugs. *NSD1* gene mutations cause Sotos syndrome (OMIM 117550), a rare autosomal dominant disorder characterized by four main features: (1) dysmorphic facial features (e.g., macrocephaly, prominent forehead, high hairline, downslanting palpebral fissures, long narrow face, pointed chin, sparse frontotemporal hair); (2) overgrowth (prenatal and postnatal accelerated growth, height and/or head circumference ≥2 SD); (3) advanced bone age; and (4) developmental delay (learning difficulties, mild to severe intellectual disability) (Luo, 2020). Case 1 exhibited high-arched palate, strabismus, micrognathia, and learning difficulties but lacked typical facial features and overgrowth, with no advanced bone age. This atypical presentation may be due to overlapping effects of the *EP300* gene.

Both cases also required differentiation from Menke-Hennekam syndrome. Menke and Hennekam analyzed 11 patients with *CREBBP* mutations and 2 with *EP300* homologous region variants, who lacked typical RSTS features but exhibited developmental delay, autism-like behaviors, short stature, and microcephaly (DDD study, 2018). Additional features included feeding difficulties, vision and hearing impairments, recurrent upper respiratory infections, and epilepsy. The two patients in this study lacked autism-like behaviors, significant feeding difficulties, recurrent infections, or epilepsy, supporting a diagnosis of RSTS based on clinical phenotypes and genetic testing.

With the widespread clinical application of new genetic technologies, an increasing number of genes have been linked to short stature. Both patients in this study presented to our pediatric clinic for growth retardation. Case 1 was initially diagnosed with Noonan syndrome (NS) due to features like downslanting palpebral fissures, webbed neck, and low hairline, along with short stature (<10th percentile), ventricular septal defect, intellectual disability, and cryptorchidism. However, genetic testing did not identify NS-associated genes but revealed pathogenic mutations in *EP300* and *NSD1*. This highlights the importance of genetic testing for accurate diagnosis and treatment in children with short stature and dysmorphic features, as well as for assessing potential disease risks. Both parents initially requested growth hormone (GH) therapy. While no literature supports GH therapy for RSTS, studies suggest a link between *EP300* mutations and gastrointestinal tumors (Wang et al., 2023). Considering potential risks, neither patient received GH therapy. Therefore, genetic testing is essential for children with short stature and dysmorphic features to clarify the etiology and guide appropriate GH therapy.

Additionally, this study compared phenotypic differences between Chinese RSTS patients with *EP300* mutations and European/American cohorts (Cohen et al., 2020), finding that arched eyebrows, downslanting palpebral fissures, and low-set ears were more common in the Chinese population. However, these features are not specific, and the statistical differences may be due to small sample sizes.

### 4.1 Study limitations

Due to the low incidence of *EP300* variants and limited reported cases, the relationship between genotype and phenotype requires further validation with larger sample sizes. In the future, we look forward to international multicenter studies and more case reports to make the phenotypic spectrum of *EP300*-related RSTS more comprehensive.

## 5 Conclusion

In summary, this study reports the clinical and genetic characteristics of two RSTS patients with *EP300* mutations, including one with dual mutations in *EP300* and *NSD1*, enriching our understanding of RSTS. Further research into the role of *EP300* gene in neuronal and skeletal cell development and the exploration of new treatments for RSTS are needed to improve early diagnosis, treatment, and prognosis.

## Data Availability

The data presented in the study are deposited in ClinVar, accession numbers SUB15286200 and SUB15286227.
